# Supplementary material for: Overcoming Sperm Cell Survival Challenges Cryopreserved in Nanoliter Volumes
Source: Int J Mol Sci. 2025 Jun 30;26(13):6343. doi: 10.3390/ijms26136343 (PMC12249527; doi:10.3390/ijms26136343)
Supplement: Supplementary file 1 [file ijms-26-06343-s001.zip › ijms-3700846-supplementary.pdf]

Overcoming Sperm Cell Survival Challenges Cryopreseved in Nanoliter Volumes

B-S Galmidi[1], Raoul Orvieto[2], Naomi Zurgil[1], Mordechai Deutsch[1], Dror Fixler[3]

1. The Biophysical Interdisciplinary Jerome Schottenstein Center for the Research and Technology of the Cellome, Physics Department, Bar-Ilan University, Ramat-Gan 5290002, Israel.
2. Department of Gynecology and Fertility, Sheba Medical Center, Tel HaShomer, Ramat Gan, 52621, Israel
3. Faculty of engineering, and The Institute of Nanotechnology and Advanced Materials, Bar Ilan University, Ramat Gan 5290002, Israel

Supplementary Material

Part S1. Volume of a droplet estimated by its cross-sectional area and contact angle

It is very difficult, if not impossible, to use a micropipette to inject volumes smaller than 0.1 μL with a satisfactory level of accuracy. However, the volume of a droplet on a surface can be accurately estimated from its cross-sectional area (A) and contact angle (α) using the geometric relations in Fig. S1.

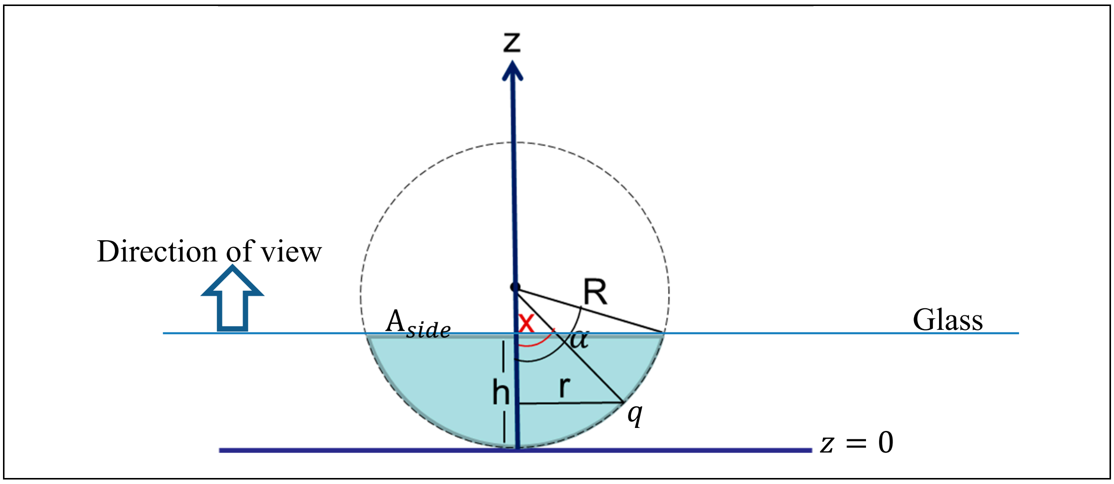

Figure S1. Calculation of the volume of a truncated sphere with radius R and maximum height of the spherical cap h (along the Z axis). The variables x and r depend on point q. A is the cross-sectional area.

The relation between the droplet radius R and its cross-sectional area A is

$$r = R \cdot \sin(x) \Longrightarrow A = \pi(R \cdot \sin(x))^2 \Longrightarrow R = \sqrt{A/\pi \cdot \sin^2(x)}$$

$$z\ (of\ point\ q) = R - R \cdot \cos(x)$$

$$dz = R \cdot \sin(x)\, dx \qquad S1$$

The volume of a spherical truncated droplet is the integral of the cross-sectional area along the z-axis:

$$V = \int_0^h \pi r^2 dz = \int_0^\alpha \pi R^2 \sin^2(x) \cdot R \cdot \sin(x)\, dx = \pi R^3 \int_0^\alpha \sin^2(x) \sin(x) dx \qquad S2$$

Using the relation $\sin^2(\theta) + \cos^2(\theta) = 1$, this integral becomes:

$$V = \pi R^3 \left(\int_0^\alpha \sin(x)dx - \int_0^\alpha \sin(x)\cos^2(x)dx \right) \qquad S3$$

Using integral tables (CRC Handbook of Chemistry and Physics, 81st Edition, Editor-in-Chief David R. Life, CRC Press, Boca Raton, FL. pp. A15) specifically $\int \sin(ax)\,dx = -\frac{1}{a}\cos(ax)$ and $\int \sin(ax)\cos^m(x)dx = -\frac{\cos^{(m+1)}(ax)}{(m+1)a}$ – Eq. 3 can be evaluated:

$$V = \pi R^3 \left(-\cos(x)|_{x=0}^{\alpha} + \frac{\cos^3(x)}{3}\bigg|_{x=0}^{\alpha} \right) = \pi R^3 \left(-\cos(\alpha) + \frac{\cos^3(\alpha)}{3} + \frac{2}{3} \right) \qquad S4$$

Inserting the relation for R from Eq. S1 yields:

$$V = \frac{\frac{1}{3}\cos^3(\alpha) - \cos(\alpha) + \frac{2}{3}}{\sqrt{\pi} \cdot \sin^3(\alpha)} A^{3/2} = f(\alpha) A^{3/2} \qquad S5$$

The contact angle (α) of 51° at room temperature of the droplet under oil on a glass surface was measured with an OCA20 goniometer (DataPhysics Instruments GmbH, Filderstadt, Germany). Thus,

$$f(\alpha = 51°) \approx 0.145 \qquad S6$$

and, finally, the relation between the volume of a droplet and its cross-sectional area is:

$$V(A) \approx 0.145 \cdot A^{3/2} \qquad S7$$

The cross-sectional area of the droplet was measured using software provided with the Olympus IX81 inverted microscope.

To examine the reliability of the model in Equation S7, precise volumes (0.1-10.0 µL) of distilled water were injected under oil on a Petri dish, and their cross-sectional areas (A) were measured using the microscope software. The droplet volume versus the cross-sectional area is plotted in Fig. S2. A power relation of $V(R) = 0.1432 \cdot A^{1.5753}$ was found with an R^2 close to unity. This is reasonably close to the relation in Equation S7, demonstrating the high effectiveness of the proposed calculation method for very small volumes of liquids.

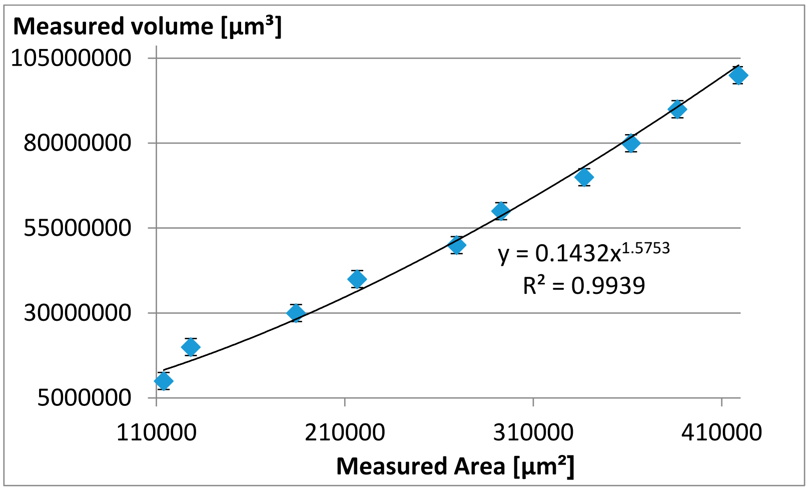

Figure S2. Plot of the volume of a droplet (V, µm^3) versus its cross-sectional area (A, µm^2).
The solid curve is the power-relation trendline calculated by Excel. The bars represent one standard deviation (SD).

Part S2. Fluorescence polarization measurements of medium droplets

To support this hypothesis, we measured the fluorescence polarization of fluorescent droplets, specifically aqueous glycerol solutions of fluorescein and various salts. The idea is that diffusion of water from the droplet would increase its viscosity and, consequently, its fluorescence polarization. Fluorescent droplets were injected under oil and their measured initial polarization was 0.041. After 180 minutes the fluorescence polarization was measured again (Fig. S3). The observed increase in fluorescence polarization with decreasing volume demonstrates the viability of our hypothesis.

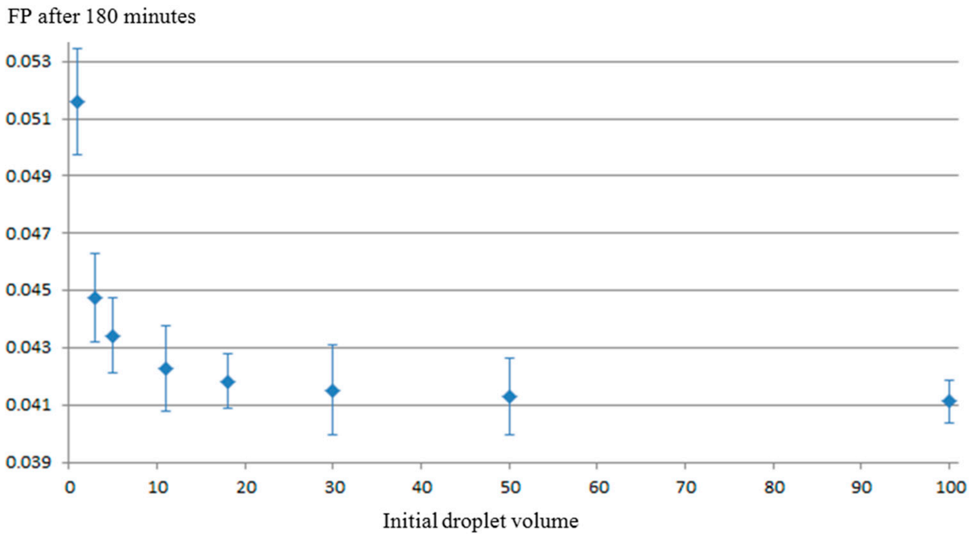

Figure S3 Fluorescence polarization (FP) measurements of fluorescent droplets after 180 minutes, as a function of the initial volume of the droplet. The initial polarization was 0.041.

Part S3. Loss of volume of distilled water droplets by diffusion into oil

Another way to observe this diffusion phenomenon is to observe with bright field microscopy, droplets of distilled water injected onto a glass plate under the oil at room temperature. The images of the droplets were acquired at 5-minute intervals for 120 minutes, and the cross-sectional area was measured; a typical before and after image of a droplet is shown in Fig. S4. Also shown is the cross-sectional area as a function of time for three typical droplets. This is a clear indication that water is diffusing from the droplet into the surrounding oil. The three curves have the same slope, indicating that the rate of shrinkage or diffusion (*i.e.*, $\frac{dA}{dt}$) is independent of the surface area; the average slope over all measurements is -88.4 ±0.08 μm^2/min.

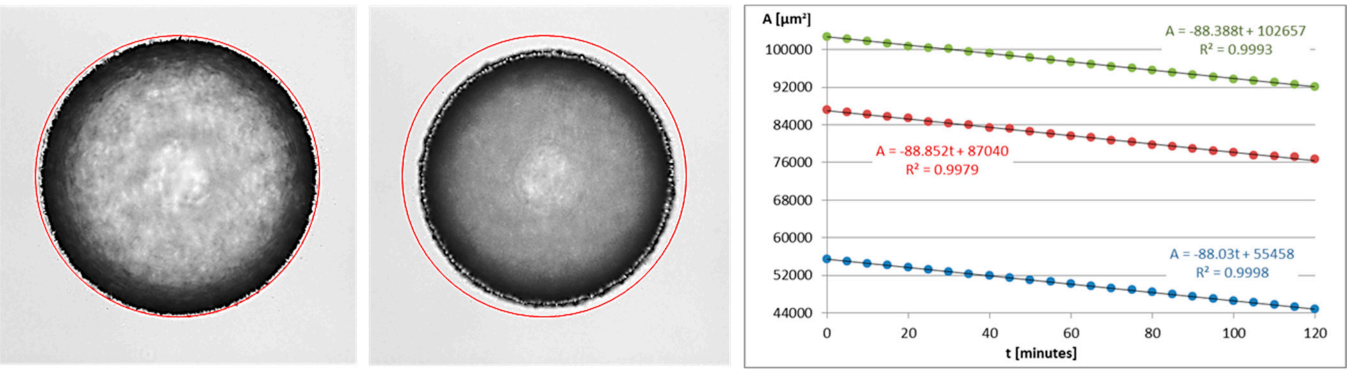

Figure S4. on the right: Cross-sectional area A as a function of time for three typical droplets. On the left: Images of initial (left) and final (right) state of shrinkage for the same droplets Photographed at X50 magnification.

Estimation of water diffusivity in oil

Distilled water was injected into Petri dishes with different diameters (d). Then, oil was poured on the water droplet, creating an oil layer with height (h). The latter was calculated by dividing the oil volume by its surface area (s). The dishes were placed on a digital analytical scale (Mettler BB244 DeltaRange, Columbus, OH, USA) with a resolution of 10^{-3}g, in an air-circulated, low-humidity room at 25°C.

For calculation, the concentration of water in the oil layer (C_h) was assumed to be null during the entire process of water loss, as any water molecule that reached the oil-air interface immediately evaporated due to the low humidity and air circulation.

Measurements were performed every 24 hours over a period of 8 days, yielding that loss of water weight is linear with time.

Next, Fick's First Law states that diffusion of molecules is due to a concentration gradient (Fick 1855) and demonstrates that:

$$j = \frac{I}{s} = -D\frac{dC}{dz} \qquad S8$$

where j is the surface density rate of water diffusion into the covering media (oil), I is the water molecule stream through the area (s) of the water-oil interface and D is the diffusion coefficient [m^2/sec].

Integrating the two right expressions of equation [1] yields:

$$\int_0^h \frac{I}{s} dz = -\int_{C_0}^{C_h} \mathrm{DdC} \qquad S9$$

where $C_h \to 0$ *and* C_0 is the concentration of water in oil immediately above the water-oil interface, i.e., the maximum capacity of water absorption in oil at room temperature or, the solubility of water in oil [kg/m^3]. Solving Equation S9 yields:

$$D \cdot C_0 = \frac{I \cdot h}{S} \qquad S10$$

so that by finding the value of I, that is to say, the rate of water loss $[\frac{kg}{h}]$, one can calculate the constant product DC_0.

The experimental results of water loss as a function of time for the 4 arrangements are presented in Fig. S5 (see upper caption of each sub-figure). The corresponding data is summarized in Table 1, from which the product $D \cdot C_0$ was extracted. The slopes in Fig. S6 are equal to I. Calculation of $D \cdot C_0$ for each of the results is shown in Table 1. The average value of said product is $\overline{D \cdot C_0} = 6.78\text{E} - 04 \pm 3.43\text{E-}05\ \text{g/m} \cdot \text{h}$.

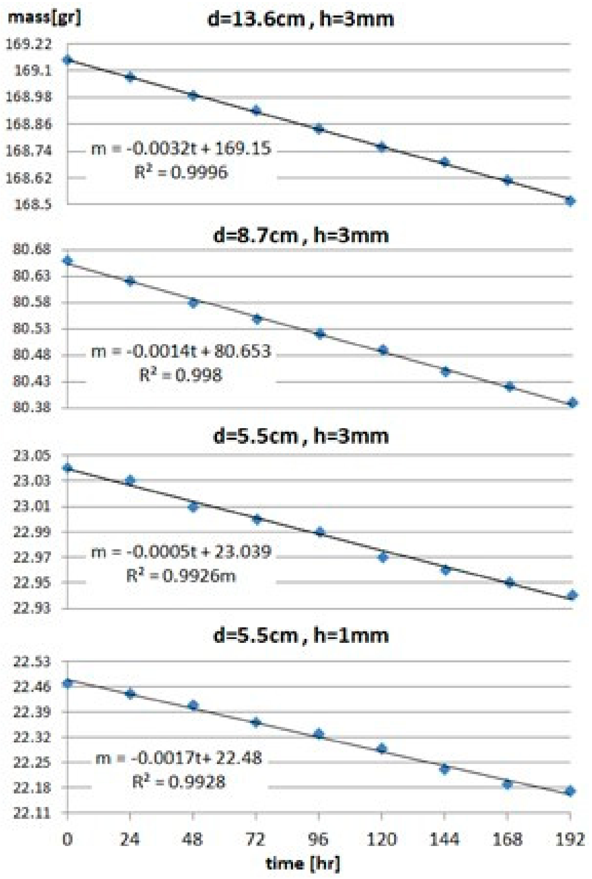

Figure S5. Rate of water loss. Results of the measurements for four different setups, where d refers to dish diameter and h to the height of the oil layer. For details, see sub-figure headings.

Control measurements were also conducted with oil alone. The results, which clearly indicate a negligible decrease in oil mass, are shown in Fig. S6.

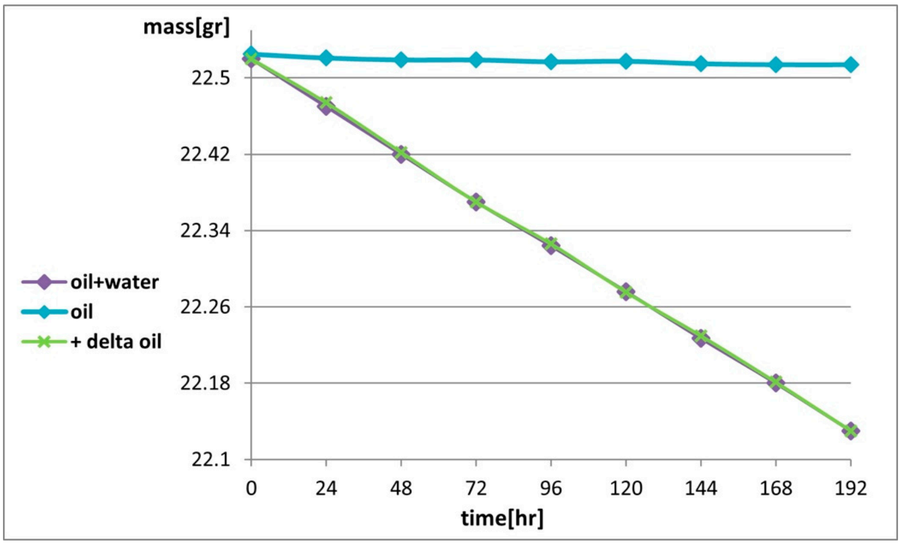

Figure S6. Evaporation time dependency. The evaporation time dependency of oil (control, blue), rate of water evaporation after covering it with a layer of oil (purple-green line). The difference between the two original lines is marked by green and is negligible.

$s[m^2]$	$h[m]$	I[gr/hr]	$DC_0[\frac{g}{m \cdot hr}]$
1.45E-02	0.003	0.0032	6.62E-04
5.94E-03	0.003	0.0014	7.07E-04
2.38E-03	0.003	0.0005	6.30E-04
2.38E-03	0.001	0.0017	7.14E-04

Table S1. Values of $D \cdot C_0$ depicted in Fig. S5. The average value was 6.78E-04 $\frac{\mathrm{g}}{\mathrm{m \cdot hr}}$

Part S4. Preventing sperm cell dehydration in nanoliter volumes.

As can be seen in Fig. S7, the use of the saturated oil results in remarkable improvements in the velocity of sperm after 30 minutes in the freezing medium and in motility after a freeze–thaw cycle.

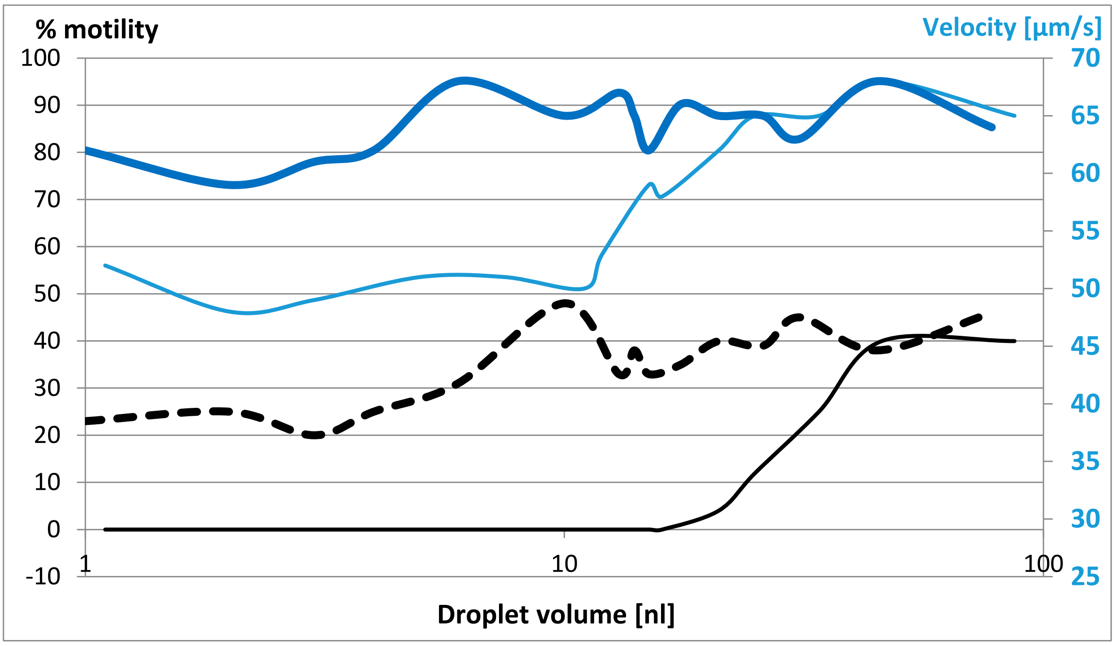

Figure S7. Velocity after 30 minutes (solid line, right axis) and normalized motility (dashed line, left axis) of sperm cells in saturated oil (thick line) and pure oil (thin line, from Fig. 1 in the manuscript) as a function of droplet volume.
